# Dietary patterns of >30,000 adolescents 9–15 years of age in rural Bangladesh

**DOI:** 10.1111/nyas.14207

**Published:** 2019-08-12

**Authors:** Andrew L. Thorne‐Lyman, Saijuddin Shaikh, Sucheta Mehra, Lee S.F. Wu, Hasmot Ali, Kelsey Alland, Kerry J. Schultze, Maithilee Mitra, Jinhee Hur, Parul Christian, Alain B. Labrique, Keith P. West

**Affiliations:** ^1^ Center for Human Nutrition, Department of International Health Johns Hopkins Bloomberg School of Public Health Baltimore Maryland; ^2^ The JiVitA Project Gaibandha Bangladesh; ^3^ Bill and Melinda Gates Foundation Seattle Washington

**Keywords:** adolescent, dietary patterns, stunting, diet, underweight, overweight, socioeconomic status, nutrition, Bangladesh, rural

## Abstract

Little is known of the usual food intakes of rural adolescents in South Asia. This study describes dietary patterns, based on >91,000 7‐day food frequencies among 30,702 girls and boys, aged 9–15 years in rural northwest Bangladesh. Three intake assessments per child, taken across a calendar year, were averaged to represent individual annual intake patterns for 22 food groups. Latent class analysis was used to assign individuals to dietary patterns based on class membership probabilities. The following five dietary patterns (class membership probabilities) were identified: (1) “least diverse” (0.20); (2) “traditional” (0.28); (3) “low vegetable/low fish” (0.23), (4) “moderately high meat” (0.20); and (5) “most diverse” (0.09). The least diverse pattern had the lowest median consumption of most foods and traditional had a relatively higher intake of most vegetables and fish. The most diverse pattern consumed both healthy and processed foods much more often than other patterns. The two most diverse patterns (4 and 5) were associated with higher socioeconomic status, body mass index, height‐for‐age Z‐score, and male gender, and the least diverse pattern showed inverse associations with these characteristics. The most diverse pattern may represent an early wave of the nutrition transition in rural Bangladesh.

## Graphical abstract

This study describes dietary patterns, based on >91,000 7‐day food frequencies among 30,702 girls and boys, aged 9–15 years in rural northwest Bangladesh. Three intake assessments per child, taken across a calendar year, were averaged to represent individual annual intake patterns for 22 food groups. These were then used to generate dietary patterns.

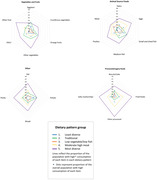

## Introduction

Interest in adolescent nutrition and health has expanded rapidly in recent years, in part due to the realization that investing in adolescent health, nutrition, and well‐being may accelerate progress toward the Sustainable Development Goals.^1^, ^2^ Adolescence may also represent a second “window of opportunity” to recover height deficits accrued early in life, as the adolescent growth spurt contributes to approximately 15–25% of adult height.^2^

During adolescence, children gain greater autonomy in decisions related to food purchase and consumption and greater exposure to food environments outside of the home.^3^ Understanding the factors that influence dietary behavior during the transition from childhood to adulthood can help inform the development of effective interventions to establish positive dietary practices that may improve nutrition and health outcomes later in the life cycle.^4^

The dietary intake and nutritional status of adolescent girls is particularly important in South Asia due to the relatively young age of pregnancy. In Bangladesh, the proportion of girls who marry and give birth prior to age 18 may be as high as 40%,^5^ raising concerns about heightened risks of adverse birth outcomes and detrimental effects on a young woman's remaining growth potential.^6^ Children born to adolescents tend to exhibit poorer growth than infants born to adult mothers.^7^ Undernutrition among adolescent girls is common in Bangladesh, with an estimated third of girls aged 15–19 years having a body mass index (BMI) <18.5 kg/m^2^.^8^, ^9^ Although the current prevalence of most micronutrient deficiencies in this age group in rural Bangladesh is uncertain, earlier work suggested the importance of multiple micronutrient deficiencies in the etiology of anemia among adolescent girls.^10^, ^11^ More recent work has suggested that the diets of adolescents in Bangladesh are low in both dietary diversity and micronutrient content.^8^, ^12^, ^13^

Dietary patterns analysis is an approach to the analysis of dietary intake data that has been increasingly used in high‐income countries, but has not yet been applied widely in low‐ and middle‐income countries (LMICs). It has been argued that this approach may be advantageous because it describes the whole of the diet, rather than individual dietary components, and can, therefore, better represent intercorrelations between different nutrients or dietary components as well as interactions of relevance to health or nutritional status outcomes.^14^ Dietary patterns analysis is often conducted in two ways: *a priori* analysis aims to examine the extent to which individuals in a population adhere to a fixed dietary consumption pattern (examples include the Mediterranean diet or the American Healthy Eating Index).^14^, ^15^ Alternatively, an *a posteriori* approach, as used in this paper, seeks to categorize individuals into different dietary pattern groups based on their existing consumption patterns of food items. Among the various approaches used for dietary patterns analysis, latent class analysis (LCA) is an increasingly used approach because it enables the direct adjustment for potential covariates and facilitates the grouping of individuals into mutually exclusive and distinct dietary patterns.^16^, ^17^, ^18^

Our present study was conducted in a large sample of more than 30,000 rural adolescents aged 9–14 years old in Northwest Bangladesh, as a follow‐up study of children whose mothers had participated in a large antenatal, weekly vitamin A or β‐carotene supplementation trial.^19^, ^20^ As part of the follow‐up study, dietary and other seasonal data were collected at three different times over a calendar year with the aim of estimating an average usual intake over a 12‐month period in a rural setting, where food availability and dietary intakes may be markedly influenced by seasonality, an important feature in rural settings in Bangladesh. The main objectives of this study were to:

1. Construct dietary patterns of adolescents and assess their prevalence in the population.
2. Examine the factors associated with these dietary patterns, including age, sex, nutritional status, and socioeconomic status (SES) measures.

## Methods

### Data collection

This study was conducted in Gaibandha District in rural northwestern Bangladesh, an area spanning 435 km^2^ across 19 administrative unions with a total population of 650,000. Participants in the study cohort were the children born to women during the JiVitA‐1 trial of vitamin A and β‐carotene supplementation in pregnancy from 2001 to 2007.^20^, ^21^ A previous follow‐up visit had been conducted in 2012 during which households agreed to future follow‐up studies. For financial reasons, a decision was made at that point to exclude from further study the population living in one administrative union who had participated in the original trial in adjacent Pirgachha *thana* in Rangpur District.^21^

From September 2015 until March 2017, 556 trained interviewers enrolled 35,056 eligible children in their birth month and administered a questionnaire, including details on household status, nutritional status, children's educational attainment, school history, and social and work activities (Fig. S1, online only). These interviewers averaged approximately 18 years of data collection experience, and quality control was assured through standardized training exercise and variable definitions, peer review of forms prior to submission, weekly meetings to discuss issues as they came up, and field rechecking when any errors were identified during data entry. Three additional visits were conducted over the course of the following year during which data were collected on diet, activity patterns, and other topics. Verbal assent was provided by all participating children/adolescents along with written parental consent.

### Variables collected

To assess diet, we used a standardized 7‐day food frequency questionnaire (FFQ) method based on an approach used successfully in other studies in Gaibandha^22^ that included 44 commonly consumed foods and beverages. These foods were selected based on focus groups conducted with the target population about what foods they commonly consumed and findings from previous dietary recalls conducted in other population subgroups in the area. Participants were asked about the number of times each food/food group was consumed in the 7 days (including holidays and weekends) prior to the interview in the presence of their mother/caretaker, who was often also the person in charge of the household kitchen. A specific question was also asked at each time point about whether or not the questionnaire had been administered during Ramadan, and if so, whether the person had fasted. Rice, the primary staple food of this population consumed at least twice daily at mealtimes, was not included due to the presumed lack of variance in the frequency of consumption within the population.

Data were collected about age, sex, current school enrollment, and whether participants engaged in different types of work for pay over the previous year. Parents were asked to report their educational attainment and categorized into three groups for the analysis (none, some primary, and grade 10 or above). Paternal occupation was examined and coded into five categories (work on own farm, day/unskilled laborer, own business, private service, and other). We also created dichotomous variables representing ownership of key assets that might be used to produce food, including homestead garden, a fish pond, fruit trees, and ownership of cattle, goats, and chickens (none, 1–2, and 3+). Household ownership of items representing SES was also collected, including bicycles, motorcycles, and TV (all treated as dichotomous any/none variables), and ownership of cellular phones (none, 1, or 2+).

Standing height was measured in the first visit to the nearest 0.1 cm using a portable locally made stadiometer, modified with a spirit level affixed to the cross‐bar to position subjects along the Frankfort plane.^23^ Weight with light clothing was measured on SECA digital scales to the nearest 100 g (SECA UNICEF Electronic Scale 890). Both measures were recorded at the first visit in triplicate and the median of the three values was used.

### Variable transformations and creation of dietary patterns

We constructed a living standards index (LSI) using principal component analysis (PCA) as described and validated in a previous paper,^24^ using variables including household ownership of 13 items and five housing characteristics. A categorical variable was then constructed representing quartiles of the LSI. Height‐for‐age Z‐scores (HAZ) and BMIZ‐for‐age were calculated using the World Health Organization growth reference for school‐aged children and adolescents.^25^ A cutoff of < –2 Z‐scores was used to define low HAZ, and BMI‐for‐age and > +2 Z‐scores was used to define high BMI‐for‐age.

In Bangladesh and many other rural settings, many foods are known to have strongly seasonal patterns. The seasonality of these foods represents a source of misclassification error when trying to characterize “normal” day to day diets using data collected during one season only.^26^ Given our goal of characterizing normal dietary patterns across the entire year, we only included participants with at least two fully completed FFQs in the analysis when constructing dietary patterns and averaged the intake from up to three recalls of each food.

LCA was chosen as the approach to characterize dietary patterns because of the desire to assign individuals to mutually exclusive and distinct dietary patterns and to adjust for Ramadan fasting. Because LCA functions better with fewer parameters, we consolidated similar foods and food groups into 22 total foods and food groups by adding the mean consumption values of similar items. Decisions about which foods to consolidate into each food group were made (1) using commonly used food groups^27^, ^28^ and similarity of nutrient content (2) maintaining distinct food items that we felt would be important to characterize given transitioning diets (such as soda) and items consumed frequently in the population (such as eggplant). A description of the food items included in each category is included in Table S1 (online only). LCA uses categorical variables as inputs, and we created variables reflecting low, medium, and high relative consumption of each item within the population using approximate demarcations at ≤20% and >80% for most foods, also presented in Table S1 (online only). For soda and tea, we created only two categories because of the large proportion of zero responses, with the higher consumption group for these foods presented in the medium consumption category. This resulted in a total of 64 diet‐related variables included in the latent class models.

### Data analysis

Proc LCA, an SAS procedure for LCA was used to undertake the latent class analyses using SAS^®^ 9.4 (SAS Institute, Cary, NC).^29^ Classification of individuals to dietary patterns was made based on estimated class membership probabilities made using LCA and item response probabilities related to the frequency of consumption of the 22 food items/groups described in Table S1 (online only). To identify the best number of classes to fit, we ran models with from two to seven latent classes. These models were freely estimated (run without parameter restrictions), using a rho stabilizing prior of 1 to improve the estimation, and at least 20 starts to find the optimal seed. To enable model convergence, we excluded participants who had any missing information on dietary variables or fasting. For five, six, and seven class models, we ran 100 starts to facilitate better identification of the optimal seed. Models were evaluated for fit using multiple criteria, including Bayesian information criterion (BIC), Akaike information criterion (AIC), and entropy, and it was found that at least seven models were needed to optimize model fit according to the BIC (Table S2, online only). From an interpretability and theoretical justification standpoint, however, it was found that models exceeding five classes led to the creation of classes representing less than 5% of the population, and that two groups were very similar in the 6‐class model. This led us to conclude that the 5‐class model had the strongest theoretical justification given our desire to identify major and distinct dietary patterns present in the population.

Following the selection of the 5‐class model, we reran the model with two additional covariates representing (1) whether or not any of the dietary recalls had been conducted during Ramadan and (2) whether the participant had fasted during at least one dietary recall. Individuals’ posterior probabilities of membership in each latent class were used to assign individuals to dietary patterns, with final assignment based on the highest posterior probability of class membership. We used radar plots to present the probability of falling into high, low, and medium consumption categories for each food given membership in each dietary pattern, and also included the proportion of the overall population falling into each consumption category as a reference point of comparison.^30^

Statistical associations between dietary score and continuous factors were tested in simple unadjusted models using one way ANOVA, and Tukey's test with Bonferroni adjustment was used for differences in groups between covariates. Chi‐squared tests were used for tests with categorical variables. *P* values < 0.05 were considered statistically significant unless otherwise noted.

### Ethical review and trial registration

The original trial was registered at clinicaltrials.gov (NCT00198822) and approval for this study was granted by the institutional review board at Johns Hopkins Bloomberg School of Public Health and by the Bangladesh Medical Research Council (BMRC).

## Results

Of the 35,056 eligible participants for the study, 30,702 were included in the analytic dataset, with exclusions due to having <2 dietary measures, incomplete diet questionnaires, or incomplete data on fasting (Fig. S1, online only). More than 99% of participants had dietary data from all three time points. About half the participants were girls, with a mean age of about 11.6 years (Table 1). Nearly, all were enrolled in school and less than 1% reported having earned money for pay outside of the household. About a quarter of participants had a BMI < –2 Z‐scores, less than 2% had a BMI >1 Z‐score, and nearly 39% had an HAZ < –2. Just over half of the mothers and fathers of participants had some education, although secondary parental education was rare. Most households had a bicycle, few had televisions, and nearly 90% had at least one cell phone. Ownership of a home garden or fruit trees was unusual, but about a quarter of households owned a fish pond.

**Table 1:** Characteristics of the population

Characteristics	*N*	*n*	Mean (SD) or %
**Adolescent characteristics**			
Mean age (years)	30,701		11.6 (1.3)
Age distribution (years)			
≤11		13,336	43.4
>11		17,365	56.6
Sex			
Boys		15,474	50.4
Girls		15,228	49.6
Currently in school (%)	30,296	28,481	94.0
BMI (Z‐score)	30,758		−1.39 (1.07)
BMI < –2 Z‐scores		8519	27.7
BMI > +1 Z‐scores		650	1.9
BMI > +2 Z‐scores		102	0.3
Height‐for‐age Z‐score (HAZ)	30,771		−1.73 (0.98)
HAZ < –2		11,909	38.7
**Parental/household characteristics**			
Maternal education			
None		12,927	42.5
Some primary		15,015	49.4
At least some secondary		2449	8.1
Paternal education			
None		14,569	48.9
Some primary		10,929	36.7
At least some secondary		4291	14.4
Paternal occupation			
Work on own farm/sharecropper		5136	17.1
Day/unskilled laborer		8988	30.0
Own business		10,781	36.0
Private service		3540	11.8
Other		1523	5.1
Household asset ownership	30,873		
Cycles		16,802	54.4
Motorcycles		2158	7.0
TV		6483	21.0
Cell phone (none)		3171	10.3
Cell phone (1)		13,529	43.8
Cell phone (2+)		14,173	45.9
Livestock ownership (any)	30,873		
Cattle		17,480	56.6
Goats		7464	24.2
Chickens			
None		8887	28.8
1–2		9741	31.6
3+		12,245	40.0
Productive agricultural assets	30,862		
Home garden		5155	16.7
Fruit trees		1227	4.0

**Figure 1:**
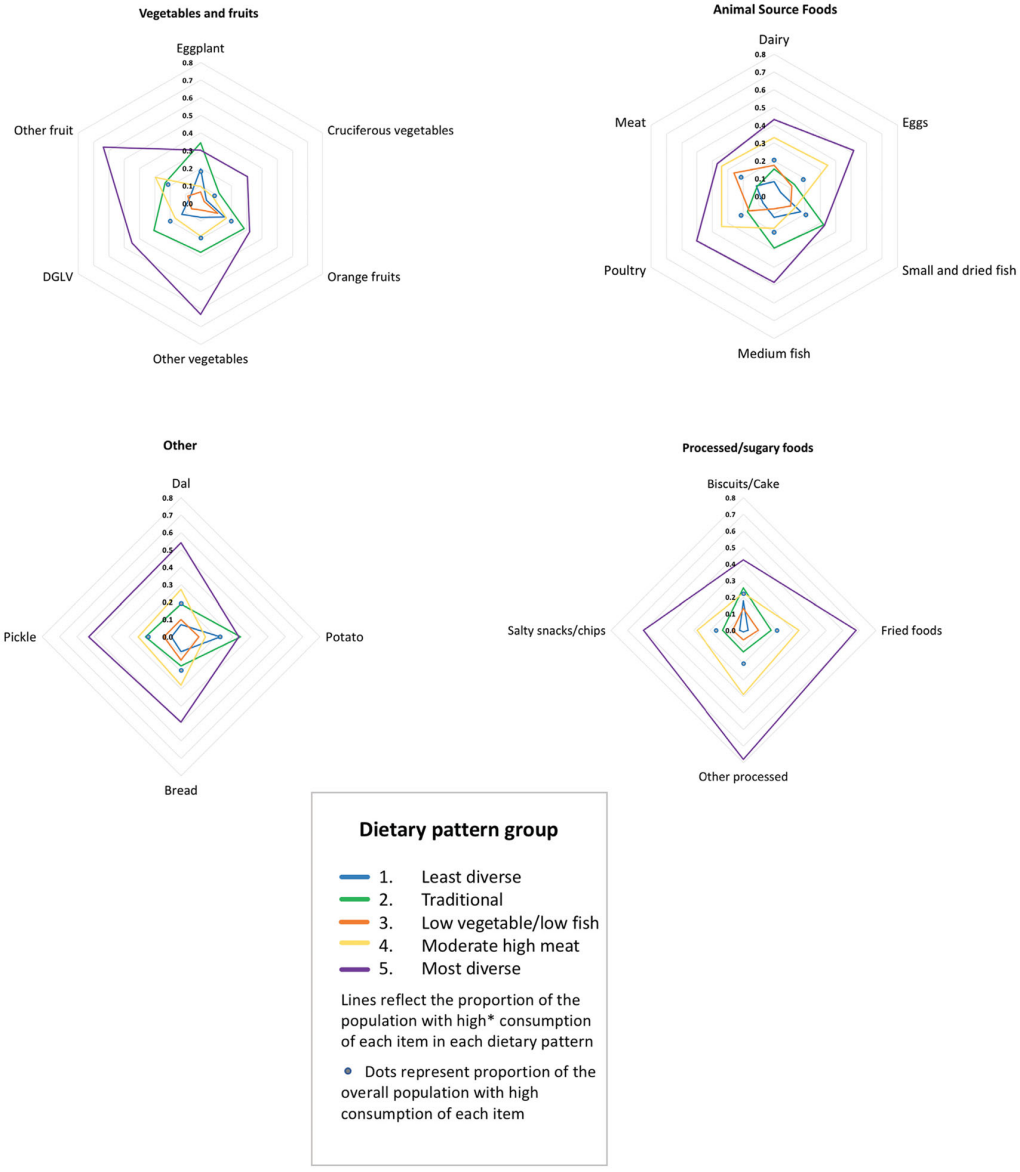
Proportion of the population in each dietary pattern consuming “high^*^” amounts of each food or food group given membership in each latent class. ^*^Dietary patterns with wider distributions represent more diverse diets. High cutoffs differ by food, and the circles represent the approximate proportion of the overall population consuming high amounts of each food, using cutoffs presented in Table S1 (online only).

**Figure 2:**
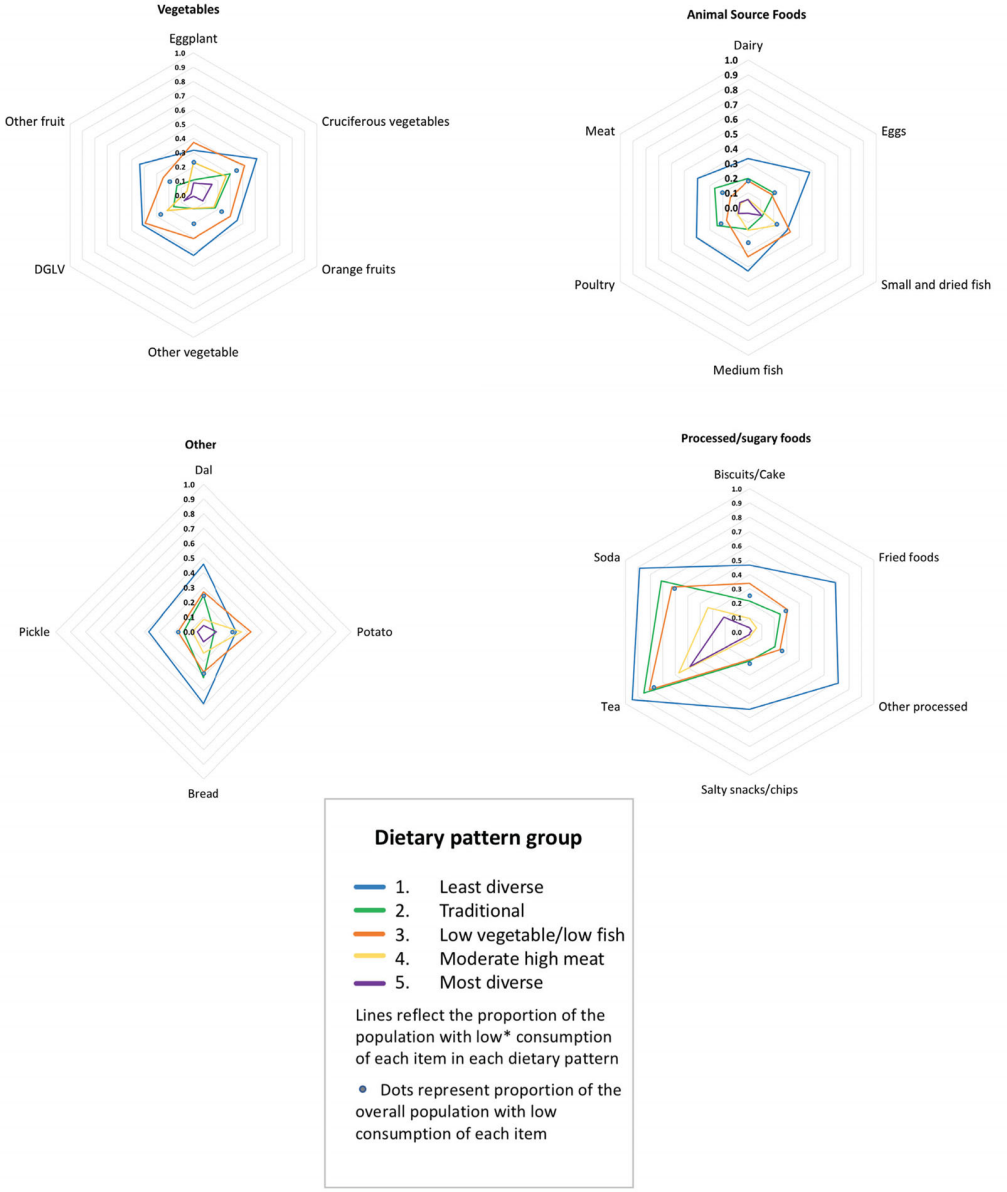
Proportion of the population in each dietary pattern consuming “low^*^” amounts of each food or food group given membership in each class. ^*^Dietary patterns with wider area distributions represent less diverse diets. Low cutoffs differ by food, and the circles represent the approximate proportion of the overall population consuming low amounts of each food, using the cutoffs presented in Table S1 (online only).

Table 2 describes each of the five dietary patterns identified through the LCA and their class membership probabilities, reflecting the proportion of the population adhering to each dietary pattern. Summaries of the distinguishing features of each dietary pattern are also provided in the table, based on examination of the data presented in Figures 1 and 2, Figure S2 (online only), and Table 3.

**Table 2:** Dietary patterns, class membership probabilities, and general description of their distinguishing characteristics

#	Name (*n*)	Probability (SE)	General description
1	Least diverse (*n* = 6210)	0.20 (0.006)	Least diverse food category characterized by low consumption of all items except for potato
2	Traditional (*n* = 8836)	0.28 (0.007)	Relatively high in eggplant, other vegetables, orange fruits, small and dried fish, and potato
3	Low vegetable and low fish (*n* = 6815)	0.23 (0.007)	Low relative dietary diversity, particularly of vegetables and fish, offset by higher consumption of meat
4	Moderate high meat (*n* = 6166)	0.20 (0.006)	Higher proportions of people in the middle distribution for many foods, and relatively high in meat, dairy, eggs, and poultry, but not fish. Relatively high in fried foods and salty snacks
5	Most diverse (*n* = 2675)	0.09 (0.003)	The most diverse dietary pattern in most food types, including other fruits, DGLV, other vegetables, all animal source foods, all processed sugary foods, and all other foods

**Table 3:** Median 7‐day frequency of intake of foods and food groups by dietary pattern (*N* = 30,702)

	1		2		3		4		5			
	Least diverse		Traditional		Low vegetable and low fish		Moderately high meat		Most diverse		Overall	
	Median	IQR	Median	IQR	Median	IQR	Median	IQR	Median	IQR	Median	IQR
Potato	11.67	7.00	16.33	6.33	14.00	7.67	12.67	7.33	16.00	6.33	14.0	7.67
Small and dried fish	3.00	3.67	6.00	5.33	3.67	5.33	3.67	3.67	6.00	5.33	4.33	5.00
Other fruit	3.00	2.50	4.00	3.33	2.00	2.67	4.67	3.33	7.33	4.33	3.67	3.33
Other vegetables	2.67	2.00	4.33	3.33	2.00	2.67	4.00	3.00	7.33	4.00	3.67	3.33
Eggplant	2.00	2.67	5.00	5.00	2.67	4.33	3.00	3.00	4.67	4.67	3.33	4.33
Medium/large fish and prawns	2.00	2.00	3.33	3.67	1.67	2.33	3.00	2.33	5.33	4.00	2.67	3.00
Other highly processed foods	2.33	1.67	2.67	2.33	1.00	0.67	4.33	2.67	6.67	3.67	2.67	3.00
Biscuits or cake	2.00	2.67	3.00	3.67	1.33	4.00	3.00	2.67	4.33	3.00	2.67	3.67
Lentils	2.00	1.67	2.00	2.00	1.33	1.67	2.67	2.00	4.00	3.00	2.00	2.00
Fried foods	1.33	1.33	1.67	1.67	0.33	1.00	2.33	2.00	4.00	2.33	1.67	2.00
Eggs	1.67	1.33	1.67	1.33	1.00	1.00	2.67	2.00	3.33	2.33	1.67	1.67
Provitamin A rich fruits	1.00	2.33	2.33	6.33	1.00	3.00	2.00	3.33	3.33	6.00	1.67	3.83
Dark green leafy vegetables	1.00	1.00	1.67	1.67	1.00	1.00	1.33	1.00	2.33	1.67	1.33	1.67
Dairy	1.00	2.33	1.00	2.00	0.33	1.67	2.33	3.00	2.67	3.33	1.00	2.33
Meat	1.33	2.67	0.67	1.33	0.33	1.00	1.67	3.00	2.00	3.00	1.00	2.00
Poultry	0.67	1.00	0.67	1.33	0.33	1.00	1.33	1.67	2.00	2.00	1.00	1.33
Cruciferous vegetables	0.33	1.00	0.67	1.33	0.00	0.67	0.67	1.33	1.33	2.00	0.67	1.33
Salty snacks	0.67	0.67	0.67	1.00	0.00	0.33	1.33	1.33	2.33	2.00	0.67	1.00
Pickle	0.67	0.67	1.00	1.33	0.33	0.67	1.00	1.00	2.00	2.00	0.67	1.17
Wheat bread	0.67	1.00	0.67	1.33	0.33	0.67	1.00	1.67	2.00	2.00	0.67	1.33
Soda	0.00	0.33	0.00	0.33	0.00	0.00	0.33	0.67	0.67	1.00	0	0.33
Tea	0.00	0.00	0.00	0.00	0.00	0.00	0.00	0.67	0.33	1.00	0	0

The “traditional” (2) and “low vegetable and low fish” (3) patterns were the two most prevalent patterns and were similar in representing relatively moderate to low dietary diversity. The traditional pattern was distinguished by having more fish and vegetables and less meat than the low vegetable and low fish pattern. About a fifth of the population fell into the “least diverse” (1) dietary pattern, characterized by distinctly lower consumption of most food items than other categories, with the exception of potato (Fig. 1). Those in the “moderately high meat” pattern (4), representing about a fifth of the population, had a tendency toward moderate consumption of many items but had a stronger tendency toward consuming meat, dairy, eggs, and poultry, rather than fish. About 9% of the population was categorized into the “most diverse” pattern (5). This pattern had a markedly greater proportion of individuals in the highest consumption category for all foods except for eggplant, potato, and small fish, which were similar in their proportions and frequency as the traditional pattern (2) (Fig. 1 and Table 3).

The most frequently consumed foods in the overall population (median/7 days) included potato (14) small and dried fish (4.33), unripened fruit (3.67), low carotenoid vegetables (3.67), eggplants (3.33), medium/large fish/shrimp (2.67), miscellaneous highly processed foods (2.67), and biscuits or a cake (2.67) (Table 3). Foods with median consumption less than once per week included salty snacks, cruciferous vegetables, wheat bread, tea, and soda.

Soda/juice and tea, both beverages typically prepared with high amounts of sugar and categorized into only two groups for variable treatment, were not frequently consumed as part of any dietary pattern, but existing consumption was largely confined to the most diverse and moderately high meat patterns, with median consumption of 0.67 and 0.33 times per week, respectively (Table 3). Those in the most diverse dietary category had a markedly higher consumption of most snack foods than other groups as well, with median consumption of other highly processed foods (6.7 times per week), fried foods (4 times per week), and biscuits/cake (4.3 times per week). Those in the least diverse dietary pattern did have some consumption of cake (2 times/week) and highly processed foods (2.3 times/week).

Only minor differences in age or age group were observed across dietary patterns (Table 4). However, clear gender differences were apparent as the majority of both those in the most diverse and moderately high meat group were boys and those in the least diverse group were girls. In contrast, the proportion of boys and girls was similar for the traditional and low vegetable and low fish groups. Further exploration of gender differences (Table S3, online only) in food group consumption suggested that greater proportions of girls consumed “low” amounts of other highly processed foods, biscuits or a cake, lentils, fried foods, salty snacks, soda, and tea compared with boys, but the opposite pattern was true for pickles. Only slight differences were apparent for most traditional food consumption (potato, fish, vegetables, eggs, dairy, and meat) by gender.

**Table 4:** Characteristics of individuals in the population (prevalence or mean)

	Dietary pattern					
	1	2	3	4	5	
	Least diverse	Traditional	Low vegetable and low fish	Moderately high meat	Most diverse	*P* value*d*
**Adolescent characteristics**						
Mean age (years)	11.7*b*	11.6*a* ^,^ *b*	11.6*a*	11.6*a*	11.6*a* ^,^ *b*	<0.001
Age distribution						
≤11 years	41.7	43.5	44.2	44.4	43.0	
>11 years	58.3	56.5	55.8	55.6	57.0	0.02
Sex						
Boys	41.8	49.1	52.0	57.4	54.4	
Girls	58.2	50.9	48.0	42.6	45.6	<0.0001
Height‐for‐age Z‐score (HAZ)	−1.85*a*	−1.81*b*	−1.71*a*	−1.58*c*	−1.55*c*	<0.0001
HAZ < −2	43.9	41.5	37.6	33.4	32.8	<0.0001
Body mass index (BMI) for age Z‐score	−1.43*a*	−1.44*a*	−1.41*a*	−1.32*b*	−1.31*b*	<0.001
BMIZ < −2	28.1	28.5	28.4	26.2	26.8	0.012
BMIZ >2	0.2	0.2	0.3	0.5	0.6	<0.0001
Currently in school (%)	94.8	95.3	93.3	94.7	94.8	<0.0001
Working for pay (%)	0.58	0.65	0.63	0.70	0.49	0.81
**Parental/household characteristics**						
Maternal education						<0.0001
None	55.2	45.3	41.7	31.5	30.3	
Some primary	42.8	49.8	51.2	52.8	52.3	
At least some secondary	2.0	4.8	7.1	15.7	17.4	
Paternal education						<0.0001
None	62.1	53.3	48.2	35.8	35.1	
Some primary	32.4	36.3	38.4	40.0	38.0	
At least some secondary	5.5	10.5	13.5	24.2	27.0	
Father occupation						<0.0001
Work on own farm/sharecropper	16.3	17.4	18.5	17.9	16.6	
Day/unskilled laborer	43.9	33.2	28.7	18.9	17.2	
Own business	29.9	36.4	35.0	39.5	43.3	
Private service	6.2	8.7	12.3	17.7	16.3	
Other	3.8	4.4	5.5	6.0	6.6	
Household asset ownership						
Cycles	47.3	54.0	55.6	60.5	62.3	<0.0001
Motorcycles	1.9	4.5	6.2	12.8	15.6	<0.0001
TV	10.5	16.3	20.4	32.7	34.5	<0.0001
Cell phone (none)	15.5	10.1	10.5	7.1	5.6	<0.0001
Cell phone (1)	51.2	47.0	44.2	37.0	33.8	<0.0001
Cell phone (2+)	33.3	42.9	45.3	56.0	60.7	<0.0001
Livestock ownership						
Cattle (any)	55.3	57.4	57.6	58.5	58.7	<0.01
Goats (any)	22.7	24.8	24.9	24.9	25.3	<0.01
Chickens						
None	31.1	29.0	27.3	25.4	26.7	<0.0001
1–2	33.9	32.8	31.6	30.2	28.3	
3+	35.1	38.3	41.1	44.5	45.0	
Productive agricultural assets						
Home garden	12.2	16.8	15.8	21.0	21.5	<0.0001
Fruit trees	2.3	3.4	2.6	5.8	6.7	<0.0001
Fish pond	20.1	25.8	27.1	33.1	34.7	<0.0001
Living standards index						
Bottom quintile	30.2	21.8	17.8	12.0	10.8	<0.0001
Top quintile	7.6	14.8	18.8	34.2	37.1	<0.0001

*^a–c^* Dietary patterns with similar superscripts were not statistically different using a post‐hoc Tukey test with Bonferroni adjustment.

*^d^*Chi^2^ for percentages and ANOVA for means comparing different dietary patterns.

Adolescents adhering to either the most diverse or moderately high meat patterns had higher mean HAZ (by about 0.25 SD) and a lower prevalence of low HAZ (by about 10%) than those following the in the least diverse traditional patterns, with those in the low vegetable and fish pattern falling in between (Table 4). The mean BMI‐for‐age Z‐score of those with the most diverse or moderately high meat patterns was approximately 0.1 SD greater than other groups, which were similar to one another, and the prevalence of low BMI < –2 Z‐scores exhibited slight differences in the same direction.

Strong and consistent relationships were observed across multiple indicators of household SES and dietary patterns. Maternal and paternal education were higher among adolescents in the most diverse and the moderately high meat dietary patterns than the other patterns, and remarkably similar to one another. Rates of any parental education (either maternal or paternal) were lowest among those in the least diverse dietary pattern. Similarly, the prevalence of children with fathers who worked as day/unskilled laborers was highest in the least diverse dietary pattern, and the proportion of those with a father owning a business or working in private service were also lowest in that group. Ownership of key assets was also lowest in the least diverse group, including cycles, motorcycles, television, and cell phones, and highest among those in the moderately high meat and most diverse groups, and similar patterns were observed for the LSI.

## Discussion

Although the diets in this large population of 9‐ to 14‐year‐olds in a rural area of Bangladesh generally lacked diversity, we identified five distinct dietary patterns within the population. These patterns exhibited consistency in their relative position across multiple measures of SES and nutritional status. The two most diverse usual dietary patterns were associated with a lower prevalence of stunting and low BMI‐for‐age, and a higher prevalence of high BMI‐for‐age, while the opposite was true for the dietary pattern with the lowest diversity. Boys were more likely to adhere to the two most diverse food patterns (4 and 5), while girls were more likely to adhere to the least diverse pattern (1). These findings suggest the potential relevance of dietary patterns analysis as a new approach to characterizing the nutrition and health of adolescents in rural Bangladesh.

Dietary patterns were differentiated from one another both by the degree of their overall diversity and by specific food items consumed. Similar to other dietary assessments conducted in Bangladesh,^31^, ^32^, ^33^ we found that fish, which we treated as two separate categories, was by far the most frequently consumed animal source food in the food system, and likely a major contributor to micronutrient and fatty acid intake of the population given relatively high nutritional value of small fish in particular.^33^, ^34^ Overall, the consumption frequency of animal source foods, including dairy in our study, was similar to findings from a recent study of adolescent pregnant women in neighboring Rangpur/Dinajpur that also used a 7‐day FFQ, but consumption of green leafy vegetables was lower in our study.^12^ This may be explained by the seasonal nature of consumption and production of these foods and our approach of averaging consumption across seasons.

Many studies have documented rising consumption of energy‐dense snack foods, highly processed foods, and sugar‐sweetened beverages as a trend contributing to the emergence of overweight and obesity in LMICs.^35^, ^36^ The overall population only consumed highly processed foods, biscuits/cake, and fried foods, a median of about two to three times each per week for each of these categories, but children identified as consuming the most diverse pattern had more frequent consumption of such items. These children also tended to come from households with relatively high SES. These findings suggest that potential targeting of such households for nutrition communication may be a helpful strategy for preventing diet‐related diseases.

Our findings also suggest the presence of gender bias in dietary quality favoring boys, supported by observations that boys made up a greater proportion of both of the most diverse dietary patterns and a lesser proportion of the least diverse dietary pattern. This may be driven in part by greater consumption of snack foods by boys. Few studies have explored differences in the diets of boys and girls among older children/adolescents in Bangladesh. A recent study found the higher probability of inadequate energy or iron intakes among adolescent girls versus boys, but found the reverse was true for BMI Z‐scores and zinc intakes.^8^ Further exploration of gender differences in the diets of adolescents is needed.

The consistent patterns observed between dietary patterns and multiple indicators of SES lend support to the validity of the approach used to generate dietary patterns and suggest that in rural Bangladesh, adolescent diets are influenced greatly by household wealth. The two most diverse patterns tended to be more common among children from wealthier and more educated households and conversely, the least diverse food pattern was more prevalent among poorer households. The traditional and low vegetable and low fish diets appeared to characterize the diets of middle‐class rural Bangladeshi adolescents. The positive associations between dietary quality and measures of wealth and SES are consistent with those from a previous study among a large sample of pregnant women from the same area^31^ and a national study that measured household dietary diversity,^32^ both of which used dietary diversity scores rather than dietary patterns to assess dietary quality. A recent study also found positive associations between household expenditure and probability of adequate energy, iron, and zinc intake.^8^

With growing economic development and rising income in Bangladesh, it may be expected that more households over time would shift toward more diverse food patterns. Open questions remain about how this may affect the prevalence of undernutrition in different population subgroups, including micronutrient deficiencies and overweight/obesity and chronic disease. Our findings suggest that dietary patterns in this age group may diversify in terms of greater consumption of both healthy traditional and processed foods.

Although our assessment of diet was limited by the lack of adjustment for energy intake and reflects only 1 year of dietary data, findings that adolescents following the most diverse dietary patterns generally had better nutritional status suggest the potential importance of diet in protecting against stunting and in improving BMI‐for‐age Z‐scores. Tracking how diets change through the life cycle and their association with nutritional status and disease outcomes may provide further insights to help guide policies.

Few studies have examined the diets of adolescents in Bangladesh and their associations with nutritional status. A recent study from a neighboring area in Northwest Bangladesh found that animal source food consumption below‐median intake was significantly associated with greater likelihood of low weight among adolescents in early pregnancy, and trends toward increased risk of short stature were observed for low animal source food and dairy intake.^12^ Previous work has also shown that dietary diversity in Bangladesh is also linked to stunting among younger children, suggesting the need to explore associations between diet and nutritional status through the life cycle.^37^

This work joins a small but growing body of literature on dietary patterns in South Asia. A recent systematic review of dietary patterns analyses in India^38^ identified eight studies exploring dietary patterns. Only two of these studies explored the dietary patterns of children.^39^, ^40^ Studies of dietary patterns in Bangladesh have generally focused on older populations in relation to the risk of noncommunicable diseases and arsenic exposure/skin lesions.^41^, ^42^

Most of the literature examining dietary patterns globally, including South Asia, has used PCA or factor analysis to generate dietary patterns. Although used extensively in the psychosocial literature, LCA has not yet been used extensively in the analysis of dietary patterns. In fact, we were able to identify only one other study from South Asia that used LCA: an analysis of data from a large National Family Health Survey in India consisting of 90,180 women aged 15–49 years.^18^ As noted in the systematic review of dietary patterns in India, an advantage of LCA over PCA is that it can be used to categorize individuals based on the whole of their diet rather than identifying clusters of a few foods, and therefore is advantageous for analyses that aim to describe the dietary patterns existing within populations.^38^

Key strengths of our study include its large sample size and inclusion of both boys and girls. The collection of 7‐day FFQ data at multiple time points throughout the year allowed us to construct patterns of seasonally varying food intakes across the seasons of a full year in a food ecology, where diets are known to vary by season. However, even with three surveys for most individuals, we cannot rule out the possibility that even a more frequent data collection might be needed to fully account for random within‐person variance given the strong seasonality of certain food items in this setting. The LCA approach to dietary patterns analysis, while more analytically intensive, does appear to have advantages, including the ability to more completely characterize diets and directly adjust for factors such as fasting on Ramadan through modeling.

The approach we used to examine dietary patterns is also subject to a number of limitations. We did not measure quantity of foods consumed as part of our FFQ. We also did not include rice, a food known to account for up to 76% of energy intake in Bangladesh;^8^ however, given that virtually everybody eats rice daily, we feel that this would not have contributed meaningful variance in intake when included as part of an FFQ. Another more general limitation of dietary patterns analysis is that a number of rather subjective decisions need to be made, such as where to draw cutoffs, how many patterns to include, or how to name or interpret meaning from the patterns.^43^

Our study raises a number of potential avenues of exploration related to adolescent diet and nutrition in Bangladesh. The adolescent growth spurt has been characterized as a potential opportunity to make up for height lost in early childhood,^2^, ^44^ yet little exploration has been undertaken of how dietary patterns might be linked to the onset of puberty, growth trajectories, or the duration of the adolescent growth spurt. Less diverse dietary patterns among 9‐ to 15‐year‐olds were associated with shorter stature and low BMI, but it may also be worth examining associations with micronutrient status and other biomarkers of nutritional status. Additionally, given that early pregnancy is the norm in this setting, it may be useful to examine how dietary patterns track over the course of adolescence and into pregnancy, whether associations exist between dietary patterns and pregnancy outcomes, and whether dietary interventions may be a useful way of improving pregnancy outcomes given the high prevalence of preterm birth and small‐for‐gestational‐age documented in Bangladesh. Given that many of the processed foods and sugar‐sweetened beverages in the food system are purchased rather than produced at home, future work is needed to understand whether it is the children themselves who purchase them or their parents, and where they are consumed. Given the rural location of our study, additional work is also needed to determine the generalizability of our findings to other settings, particularly in urban contexts, where the availability of processed foods and purchasing power of this age group may both be greater.

Further methodological questions also exist related to dietary patterns analysis. Many dietary surveys in low and middle incomes have been confined to one point in time or season, and uncertainty exists around how representative dietary patterns generated from such data are of normal dietary patterns spanning across seasons or years. Additional studies with data collected over time could provide insights on how frequent data collection should be to minimize random within‐person error in the characterization of normal diets in populations with significant seasonal dietary variation.

## Author contributions

A.T.L., K.P.W. Jr., and P.C. drafted the paper. A.T.L. and L.W. conducted analysis. S.S., S.M., L.W., H.A., K.Z., K.J.S., M.M., J.H., P.C., A.L., and K.P.W. Jr. reviewed paper draft and provided comments. S.S., S.M., L.W., H.A., K.Z., K.J.S., M.M., J.H., P.C., A.L., and K.P.W. Jr. conducted research.

## Supporting information


**Figure S1**. Flow diagram of the JiVitA‐1 cohort follow‐up study and the analytic dataset for dietary patterns analysis.
**Figure S2**. Proportion of the population in each dietary pattern consuming “medium^*^” amounts of each food or food group given latent class relative to the overall population.
**Table S1**. Food groups and food items consumed and cutoffs for high and low 7‐day average intake used as inputs in the latent class analysis.
**Table S2**. Model‐fit statistics for latent class models.
**Table S3**. Comparison of the proportion of boys versus girls consuming low^*^ intake of each food group/item.
