# Supplementary material for: Dietary patterns of >30,000 adolescents 9–15 years of age in rural Bangladesh
Source: Ann N Y Acad Sci. 2019 Aug 12;1468(1):3–15. doi: 10.1111/nyas.14207 (PMC7318683; doi:10.1111/nyas.14207)
Supplement: Supplementary file 1 — Figure S1. Flow diagram of the JiVitA‐1 cohort follow‐up study and the analytic dataset for dietary patterns analysis. Figure S2. Proportion of the population in each dietary pattern consuming “medium*” amounts of each food or food group given latent class relative to the overall population. Table S1. Food groups and food items consumed and cutoffs for high and low 7‐day average intake used as inputs in the latent class analysis. Table S2. Model‐fit statistics for latent class models. Table S3. Comparison of the proportion of boys versus girls consuming low* intake of each food group/item. [file NYAS-1468-3-s001.docx]

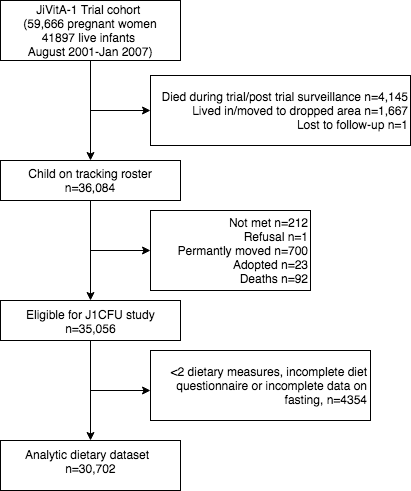


**Figure S1**. Flow diagram of the JiVitA-1 Cohort Follow-up Study and the analytic dataset for dietary patterns analysis

**Figure S2.** Proportion of the population in each dietary pattern consuming “medium*” amounts of each food or food group given latent class relative to the overall population. * Wider area distributions represent a tendency towards medium dietary diversity. Cutoffs differ by food, and the circles represent the approximate proportion of the overall population consuming medium amounts of each food, using the cutoffs presented in Table S1. For soda and tea, cutoffs represent those consuming any consumption of these items due to dichotomous treatment of these two variables.

**Table S1.** Food groups and food items consumed and cutoffs for high and low 7-day average^a^ intake used as inputs in the latent class analysis

|  |  | Usual^a^ intake frequency per week | |
| --- | --- | --- | --- |
| Food Group/item | Included foods | Low^b^ cutoff | High^b^ cutoff |
| Potato |  | < 9 | > 18.4 |
| Fish, small, dried | Small fish and dried fish | < 2 | > 8 |
| Fruit, unripened | Green fruit *shobji*^c^, guava, ripe banana, watermelon, citrus (pommello, jambura, orange), pineapple, jamur | < 1.7 | > 6 |
| Vegetables, low carotenoid | Okra, cucumber, tomato, beans, gourds | < 1.7 | > 6 |
| Eggplant |  | < 1.4 | > 6.7 |
| Fish, medium/large and prawns | Medium/large fish and shrimp/prawns | < 1.4 | > 5 |
| Processed foods, miscellaneous | Candy, mishti (sweets), noodles | < 1.4 | > 4.7 |
| Biscuits or cake |  | < 1 | > 5 |
| Lentils | *Dal*/legumes/pulses, *khichuri* | < 1 | > 3.7 |
| Fried foods |  | < 1 | > 3 |
| Eggs | Eggs (*any kind)* | <1 | >3 |
| Provitamin A rich fruits | Pumpkins, ripe papaya, jackfruit, mango | < 0.5 | > 5.4 |
| Dark green leafy vegetables | Any dark green leafy vegetables | < 0.7 | > 2.4 |
| Dairy | Milk (*any kind*), yoghurt | 0 | > 3 |
| Meat | Red meat (Goat, lamb, beef, water buffalo), liver | 0 | > 3 |
| Poultry | Chicken, duck, pigeon | 0 | > 2 |
| Cruciferous vegetables | Cabbage, cauliflower | 0 | > 2 |
| Salty snacks |  | 0 | > 1.7 |
| Pickle |  | 0 | > 1.7 |
| Wheat bread |  | 0 | > 1.7 |
| Soda | Soda, juice packets, sugary drinks | 0 |  |
| Tea | Milk tea or coffee | 0 |  |

^a^Intakes were averaged over three or two 7 day recalls conducted at different time points over a calendar year

^b^Cutoffs for low and high relative intake were demarcated at approximately the 20^th^ and 80^th^ percentiles of the distribution to create 3 categories for each food/food group, except tea and soda which had only 2 categories. The resulting categorical variables were then used as inputs for the creation of dietary patterns using LCA.

**Table S2.** Model-fit statistics for latent class models

|  | Number of classes (dietary patterns) | | | | | |
| --- | --- | --- | --- | --- | --- | --- |
|  | **2** | **3** | **4** | **5** | **6** | **7** |
| Log-Likelihood | -610388.43 | -605456.45 | -602954.76 | -601878.25 | -601048.23 | -600178.10 |
| G-squared | 588184.43 | 578320.47 | 573317.09 | 571164.07 | 569504.02 | 567763.76 |
| AIC | 588354.43 | 578576.47 | 573659.09 | 571592.07 | 570018.02 | 568363.76 |
| BIC | 589062.66 | 579642.98 | 575083.87 | 573375.14 | 572159.37 | 570863.39 |
| Adjusted BIC | 588792.53 | 579236.19 | 574540.44 | 572695.05 | 571342.63 | 569909.99 |
| Entropy | 0.76 | 0.73 | 0.66 | 0.64 | 0.65 | 0.63 |

**Table S3.** Comparison of the proportion of boys versus girls consuming low* intake of each food group/item

|  | Boys (%) | Girls (%) | P-value^a^ |
| --- | --- | --- | --- |
| Potato | 20.3 | 19.2 | 0.02 |
| Small and dried fish | 22.2 | 22.6 | 0.50 |
| Other fruit | 22.8 | 23.1 | 0.57 |
| Other vegetables | 19.9 | 20.1 | 0.64 |
| Eggplant | 23.1 | 23.3 | 0.68 |
| Medium/large fish and prawns | 24.4 | 23.0 | 0.004 |
| Other highly processed foods | 20.7 | 31.6 | <0.0001 |
| Biscuits or cake | 23.7 | 26.8 | <0.0001 |
| Lentils | 22.0 | 26.4 | <0.0001 |
| Fried foods | 22.1 | 36.2 | <0.0001 |
| Eggs | 19.9 | 21.5 | 0.0003 |
| Provitamin A rich fruits | 20.3 | 18.7 | <0.001 |
| Dark green leafy vegetables | 27.4 | 26.2 | 0.014 |
| Dairy | 18.2 | 17.8 | 0.43 |
| Meat | 19.6 | 21.1 | <0.001 |
| Poultry | 21.1 | 21.6 | 0.33 |
| Cruciferous vegetables | 35.3 | 34.2 | <0.05 |
| Salty snacks | 19.7 | 24.1 | <0.0001 |
| Pickle | 20.8 | 13.5 | <0.0001 |
| Wheat bread | 28.4 | 28.1 | 0.64 |
| Soda | 58.2 | 63.2 | <0.0001 |
| Tea | 71.1 | 83.4 | <0.0001 |

*Thresholds for low intake defined in Table S1
